# Benchmarking Multimodal Large Language Models for Cardiopulmonary Findings on Chest Radiographs: Sex-Stratified Discrimination and Operating Characteristics

**DOI:** 10.3390/diagnostics16132131

**Published:** 2026-07-07

**Authors:** Matteo Haupt, Arne Bischoff, Myriam Atoubi, Rohit Philip Thomas, Martin H. Maurer

**Affiliations:** Department of Diagnostic and Interventional Radiology, Carl von Ossietzky Universität Oldenburg, 26129 Oldenburg, Germany

**Keywords:** artificial intelligence, chest radiography, multimodal large language models, benchmark, sex-stratified analysis

## Abstract

**Background/Objectives**: To characterize the zero-shot diagnostic behavior of three commercial multimodal large language models (MLLMs) on cardiopulmonary chest radiograph findings and to assess sex-stratified performance differences. **Methods**: GPT-5.4, Claude Opus 4.5, and Gemini 2.5 Pro were evaluated in 4500 pathology-specific radiograph evaluations based on frontal chest radiographs from the publicly available CheXpert dataset. Three balanced cohorts of 1500 images each were constructed for cardiomegaly, pulmonary edema, and pleural effusion (375 per sex-by-label subgroup). All models received identical zero-shot prompts requesting binary classification. The primary outcome was area under the receiver operating characteristic curve (AUC-ROC) with 95% bootstrap confidence intervals. Secondary outcomes were sensitivity and specificity. **Results**: A total of 4500 pathology-specific radiograph evaluations were performed across the three cohorts (2250 male and 2250 female cohort entries; mean age 58.4 ± 18.0 years). GPT-5.4 achieved the highest discrimination (AUC-ROC 0.836–0.883) but showed very low sensitivity (0.043–0.424) with near-perfect specificity (0.977–0.997). Claude Opus 4.5 showed moderate discrimination (AUC-ROC 0.698–0.761) with balanced sensitivity (0.396–0.876) and specificity (0.461–0.863). Gemini 2.5 Pro showed moderate discrimination (AUC-ROC 0.745–0.770) but favored sensitivity (0.673–0.973) at the expense of specificity (0.241–0.804). Sex-stratified analyses showed consistently higher AUC point estimates in male patients for cardiomegaly and pulmonary edema, but smaller and less directional differences for pleural effusion. **Conclusions**: Commercial MLLMs differ considerably in operating profiles, ranging from ultraconservative to aggressive detection, so that strong aggregate discrimination can mask sensitivity too low for reliable detection. None of the evaluated models are currently suitable for autonomous chest radiograph interpretation. Sex-stratified differences were modest but non-uniform, supporting subgroup-aware reporting rather than reliance on pooled metrics alone.

## 1. Introduction

Multimodal large language models (MLLMs) are general-purpose AI systems that integrate visual and textual information [1,2,3]. In radiology, this has raised growing interest in whether commercial MLLMs can support image interpretation in zero-shot settings, without task-specific fine-tuning [4,5,6,7,8]. Chest radiography is a suitable test case because it is one of the most widely used imaging modalities and is central to diagnosing common cardiopulmonary conditions in emergency, inpatient, and outpatient care [9].

Most prior AI research in chest radiography has focused on conventional deep learning classifiers trained for specific tasks on labeled datasets [9,10,11,12]. More recently, the field has shifted toward large-scale foundational encoders that show strong feature representation across radiographic tasks [13,14]. By contrast, commercial MLLMs are increasingly accessible through application programming interfaces and may be applied to a wide range of clinical tasks [1]. These models are not approved medical devices, and the present evaluation is intended solely for research purposes. Studying how they perform nonetheless matters, because such systems are widely accessible and may be used in unsanctioned ways that carry patient-safety implications. However, their actual diagnostic behavior in radiographic interpretation remains insufficiently characterized. In particular, head-to-head comparisons of selected commercial models under identical conditions are scarce. Such comparisons are important because models with similar overall discrimination may still differ in operating profile, including the balance between sensitivity and specificity, which directly affects their potential clinical use.

Beyond aggregate performance, subgroup robustness matters before any clinical use. Prior work has shown that AI systems for medical imaging may exhibit performance disparities across demographic groups, including sex-based differences in chest radiograph interpretation [15,16,17,18]. Such disparities are clinically relevant because biased or unstable model behavior could amplify existing inequities in diagnostic pathways [19]. For this reason, performance evaluation should not be limited to pooled metrics alone but should also examine whether model behavior differs between male and female patients.

In this study, we benchmarked three selected commercial MLLMs (GPT-5.4, Claude Opus 4.5, and Gemini 2.5 Pro) for zero-shot classification of cardiomegaly, pulmonary edema, and pleural effusion on frontal chest radiographs from the CheXpert dataset [20]. We compared their overall diagnostic performance and operating characteristics under identical prompting conditions and assessed sex-stratified performance differences across pathologies. Combining a head-to-head benchmark with subgroup analysis, the study evaluates the clinical relevance and subgroup robustness of commercial MLLMs for chest radiograph interpretation.

## 2. Methods

### 2.1. Study Design

This retrospective benchmark study evaluated the diagnostic performance of three commercially available MLLMs for zero-shot classification of cardiopulmonary findings on frontal chest radiographs. Using the publicly available CheXpert dataset, we constructed three pathology-specific cohorts for cardiomegaly, pulmonary edema, and pleural effusion and compared pooled and sex-stratified model performance under identical prompting and preprocessing conditions.

This retrospective study used exclusively publicly available, anonymized data from the CheXpert dataset; institutional review board approval and informed consent were waived.

### 2.2. Dataset and Cohort Construction

CheXpert is a large-scale chest radiograph dataset collected at Stanford University Medical Center and contains radiographic examinations with labels derived from associated radiology reports [20]. For this study, we used the training partition with VisualCheXbert labels to improve label quality and agreement with radiologist interpretation [21].

Three pathology-specific analysis cohorts were constructed for cardiomegaly, pulmonary edema, and pleural effusion. Each cohort comprised 1500 frontal chest radiographs balanced across four sex-by-label subgroups: male positive, male negative, female positive, and female negative, with 375 images per subgroup. Inclusion criteria were frontal projection, binary ground-truth label for the target pathology, known sex and age, and verified image availability. Studies with co-occurring positive labels for the other two target pathologies were excluded to reduce overlap between target findings. Patient-level deduplication was applied to ensure that each patient contributed only one study per cohort. The negative subgroups for cardiomegaly and pleural effusion were identical after cohort construction and balancing, resulting in the same age summary statistics in Table 1.

Age matching between male and female groups was performed using two-bin stratification (<70 years and ≥70 years) with proportional allocation within each pathology-specific cohort. This two-bin approach was chosen to preserve sufficient sample size within each sex-by-label subgroup after pathology-specific filtering and patient-level deduplication. Projection was controlled by pathology: the cardiomegaly and pleural effusion cohorts were restricted to posteroanterior (PA) views, whereas the pulmonary edema cohort included both PA and anteroposterior (AP) views to reflect the clinical distribution of edema imaging in acute-care settings.

### 2.3. Models and Inference Protocol

Three commercial MLLMs were evaluated: GPT-5.4 (OpenAI, San Francisco, CA, USA), Claude Opus 4.5 (Anthropic, San Francisco, CA, USA), and Gemini 2.5 Pro (Google, Mountain View, CA, USA). All models were accessed through their respective commercial application programming interfaces. API calls were made between March and April 2026.

Images were preprocessed uniformly before inference. Each radiograph was converted to RGB format, resized with preservation of aspect ratio to a maximum side length of 1024 pixels, and encoded as JPEG with 95% quality. All models received the same zero-shot prompt structure: a system instruction defining the model’s role as an expert thoracic radiologist performing binary visual classification, followed by a user prompt asking whether the target pathology was present and requesting a structured JSON response containing a binary label (PRESENT or ABSENT) and an integer confidence score (0–100) (Supplementary Table S1). The confidence score was converted to a probability of pathology presence: for PRESENT labels, *p* = confidence/100; for ABSENT labels, *p* = 1 − confidence/100. This continuous probability served as the decision variable for AUC-ROC computation. No patient demographic information, including sex, was provided to the models. Temperature was set to 0 for all model calls.

### 2.4. Outcomes

The primary outcome was diagnostic discrimination, measured by the area under the receiver operating characteristic curve (AUC-ROC). Secondary outcomes were sensitivity and specificity based on the binary PRESENT/ABSENT classification returned by each model.

Performance was first assessed on the full pathology-specific cohort pooled across sexes. Additional sex-stratified analyses were then performed separately for male and female patients. Sex-based performance differences were quantified as male minus female values for AUC, sensitivity, and specificity.

### 2.5. Statistical Analysis

AUC-ROC was calculated for each model and pathology on the pooled cohort and separately within male and female subgroups. Pairwise differences in AUC-ROC between models were assessed using paired, two-sided DeLong tests, because all models were evaluated on the same images within each pathology-specific cohort. Tests were performed separately for each pathology. To account for the three pairwise model comparisons within each pathology, Holm-Bonferroni-adjusted *p* values are reported. Sensitivity and specificity were calculated from the binary classification output of each model.

Uncertainty was estimated using nonparametric bootstrap resampling with 1000 iterations. For each metric, 95% confidence intervals were derived from the bootstrap distribution. For sex-stratified analyses, differences between male and female performance were summarized as ΔAUC, ΔSensitivity, and ΔSpecificity, with corresponding 95% bootstrap confidence intervals.

## 3. Results

### 3.1. Cohort Characteristics

The study comprised 4500 pathology-specific radiograph evaluations from CheXpert, organized into three pathology-specific cohorts of 1500 images each for cardiomegaly, pulmonary edema, and pleural effusion. Each cohort was balanced across four sex-by-label subgroups, with 375 images per subgroup.

**Figure 1:**
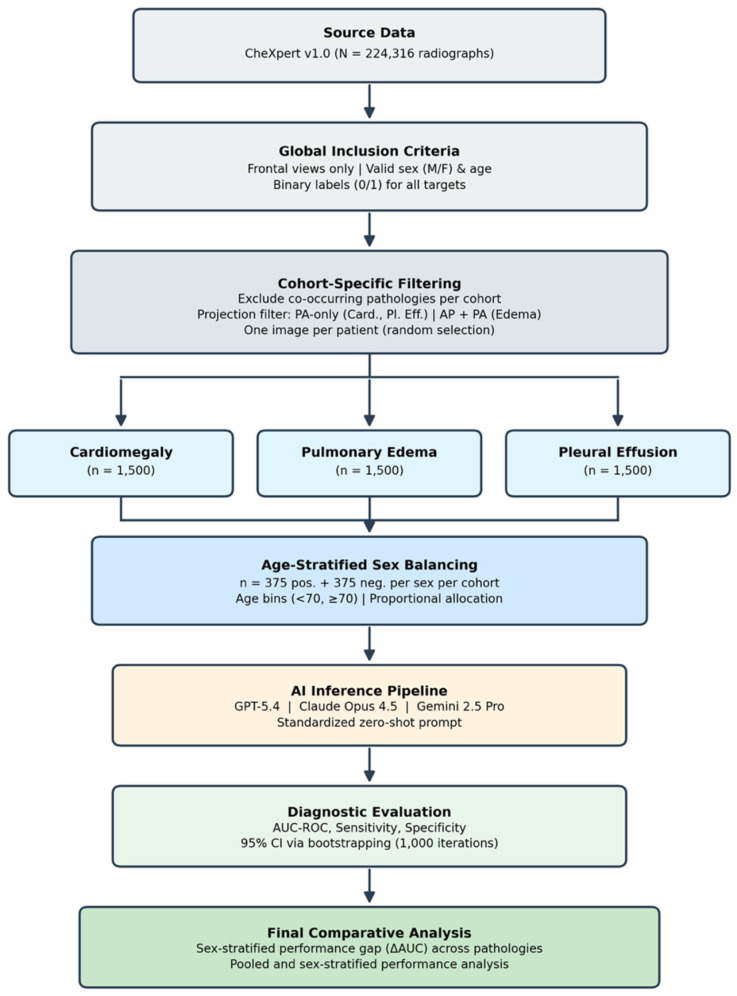
Study design and analysis pipeline. Flowchart of cohort construction, subgroup balancing, model inference, and performance evaluation. Frontal chest radiographs from CheXpert were filtered using predefined inclusion and exclusion criteria and assembled into three pathology-specific cohorts for cardiomegaly, pulmonary edema, and pleural effusion (*n* = 1500 each). Each cohort was balanced across sex and disease status (375 positive and 375 negative cases per sex). Standardized zero-shot inference was performed with GPT-5.4, Claude Opus 4.5, and Gemini 2.5 Pro, followed by pooled and sex-stratified diagnostic evaluation.

Cohort characteristics are summarized in Table 1. Age distributions were closely matched between male and female patients within each pathology and label stratum. In the cardiomegaly cohort, mean age was 63.9 years in positive female patients and 64.5 years in positive male patients, compared with 54.4 and 55.3 years in the corresponding negative groups. In the pulmonary edema cohort, mean age ranged from 57.5 to 59.6 years across subgroups. In the pleural effusion cohort, positive cases were older than negative cases, while male and female patients remained well balanced within status categories (Figure 1).

### 3.2. Overall Diagnostic Performance

Overall diagnostic performance pooled across sexes is shown in Table 2. The three models showed clearly different operating characteristics despite receiving identical images and prompts.

GPT-5.4 achieved the highest discrimination across all three pathologies, with AUC values of 0.859 (95% CI, 0.842–0.877) for cardiomegaly, 0.836 (95% CI, 0.816–0.856) for pulmonary edema, and 0.883 (95% CI, 0.866–0.899) for pleural effusion. However, this model showed a strongly conservative classification profile, with very low sensitivity for cardiomegaly and pulmonary edema at 0.293 (95% CI, 0.262–0.326) and 0.043 (95% CI, 0.029–0.058), respectively, alongside near-perfect specificity ranging from 0.977 to 0.997. Sensitivity was higher for pleural effusion at 0.424 (95% CI, 0.390–0.459), but the same general operating profile remained evident.

Claude Opus 4.5 showed lower overall discrimination than GPT-5.4, with AUC values ranging from 0.698 to 0.761, but exhibited a more balanced sensitivity-specificity tradeoff. Sensitivity ranged from 0.396 (95% CI, 0.360–0.431) for pleural effusion to 0.876 (95% CI, 0.850–0.897) for pulmonary edema, while specificity ranged from 0.461 (95% CI, 0.427–0.495) for pulmonary edema to 0.863 (95% CI, 0.837–0.885) for pleural effusion.

Gemini 2.5 Pro showed overall discrimination comparable to Claude Opus 4.5, with AUC values of 0.760 (95% CI, 0.736–0.781) for cardiomegaly, 0.745 (95% CI, 0.723–0.768) for pulmonary edema, and 0.770 (95% CI, 0.747–0.794) for pleural effusion, but exhibited the most aggressive detection profile. Sensitivity was highest among the three models for cardiomegaly at 0.916 (95% CI, 0.896–0.936) and for pulmonary edema at 0.973 (95% CI, 0.961–0.984), but this came at the cost of low specificity, particularly for pulmonary edema at 0.241 (95% CI, 0.210–0.271) and cardiomegaly at 0.449 (95% CI, 0.414–0.483). For pleural effusion, Gemini 2.5 Pro showed a more moderate balance, with sensitivity of 0.673 (95% CI, 0.641–0.709) and specificity of 0.804 (95% CI, 0.775–0.830).

**Figure 2:**
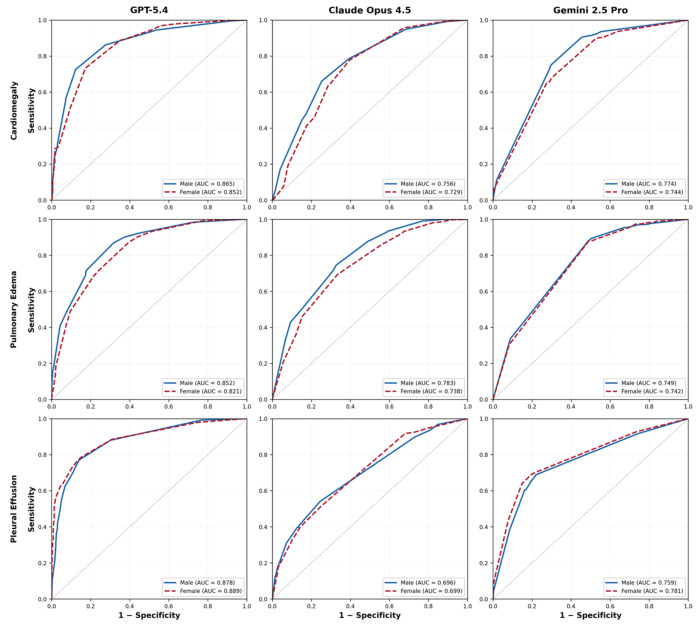
Receiver operating characteristic curves stratified by pathology, model, and sex. Each panel shows the receiver operating characteristic curve for one model-pathology combination. Solid blue lines indicate male patients, dashed red lines indicate female patients, and the diagonal gray line indicates chance performance.

Overall, the models differed widely in operating profile. GPT-5.4 prioritized specificity, Gemini 2.5 Pro prioritized sensitivity, and Claude Opus 4.5 occupied an intermediate position with the most balanced binary classification behavior (Figure 2). Pairwise DeLong testing confirmed that GPT-5.4 achieved significantly higher AUC-ROC than both Claude Opus 4.5 and Gemini 2.5 Pro for all three pathologies after Holm-Bonferroni adjustment (all adjusted *p* < 0.001; Supplementary Table S2). Differences between Claude Opus 4.5 and Gemini 2.5 Pro were not statistically significant for cardiomegaly (adjusted *p* = 0.194) or pulmonary edema (adjusted *p* = 0.162), but Gemini 2.5 Pro significantly outperformed Claude Opus 4.5 for pleural effusion (AUC 0.770 vs. 0.698; adjusted *p* < 0.001).

### 3.3. Sex-Stratified Performance Analysis

**Figure 3:**
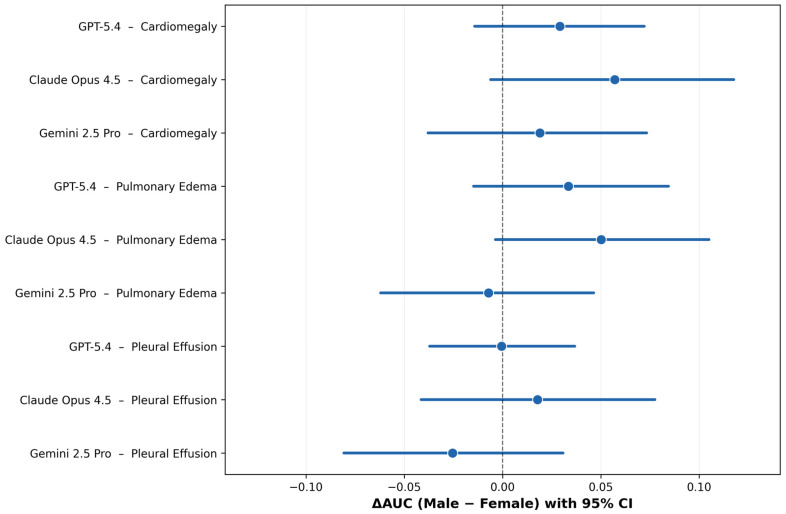
Sex-stratified differences in diagnostic discrimination across models and pathologies. Forest plot showing differences in AUC-ROC between male and female patients, defined as ΔAUC = Male − Female. Points represent estimated subgroup differences and horizontal bars represent 95% bootstrap confidence intervals based on 1000 resampling iterations. The dashed vertical line at zero indicates no difference between sexes.

Sex-stratified performance results are shown in Table 3 and Figure 2 and Figure 3. Across pathologies, subgroup differences between male and female patients were generally modest, although some model- and metric-specific deviations were observed.

For cardiomegaly, all three models showed higher AUC point estimates in male patients than in female patients. AUC was 0.865 versus 0.852 for GPT-5.4, 0.756 versus 0.729 for Claude Opus 4.5, and 0.774 versus 0.744 for Gemini 2.5 Pro, corresponding to ΔAUC values of +0.013, +0.027, and +0.030, respectively. Sensitivity differences were small for all three models. The largest subgroup difference in this cohort was for Gemini 2.5 Pro specificity, which was higher in male than in female patients (0.493 vs. F 0.405; ΔSpec, +0.088; 95% CI, +0.015 to +0.162).

For pulmonary edema, AUC point estimates were again slightly higher in male patients across all three models, with ΔAUC values of +0.031 for GPT-5.4, +0.045 for Claude Opus 4.5, and +0.007 for Gemini 2.5 Pro. Sensitivity differences remained small. The largest subgroup deviation was for Claude Opus 4.5 specificity, which was higher in male than in female patients (0.499 vs. 0.424; ΔSpec, +0.075; 95% CI, +0.004 to +0.147). GPT-5.4 also showed slightly higher specificity in male patients, although the absolute difference was small.

For pleural effusion, subgroup differences were smaller and less directional. GPT-5.4 and Gemini 2.5 Pro showed slightly lower AUC values in male than in female patients, whereas Claude Opus 4.5 showed near-identical performance between sexes. Differences in sensitivity and specificity were likewise small, and all corresponding confidence intervals overlapped zero.

Across analyses, sex-stratified results showed mostly modest differences between male and female patients, with some model- and pathology-specific deviations, particularly in specificity. Across cardiomegaly and pulmonary edema, AUC point estimates were consistently higher in male patients for all three models, whereas pleural effusion showed smaller and mixed subgroup differences (Figure 3).

## 4. Discussion

This study provides a controlled head-to-head benchmark of three selected commercial multimodal large language models for cardiopulmonary chest radiograph interpretation under identical zero-shot conditions. Two results stand out. First, the models showed clearly different operating profiles despite identical images and prompts. Second, sex-stratified analyses showed modest but non-uniform differences between male and female patients. These results indicate that evaluation of commercial MLLMs for radiologic use should go beyond aggregate discrimination and include subgroup-aware assessment of operating behavior.

Overall discrimination and binary classification behavior diverged sharply across models. GPT-5.4 achieved the highest AUC values across all three pathologies, indicating the strongest ranking performance overall. However, this discrimination was paired with an ultraconservative operating profile, characterized by extremely low sensitivity and near-perfect specificity, particularly for pulmonary edema and cardiomegaly. GPT-5.4 rarely classified cases as positive, even though its probability estimates retained substantial discriminatory information. By contrast, Gemini 2.5 Pro exhibited the opposite pattern, favoring very high sensitivity at the cost of a substantial false-positive burden, especially for pulmonary edema and cardiomegaly. This aggressive detection profile is consistent with behavior reported for medically tuned Gemini variants [22]. Claude Opus 4.5 occupied an intermediate position, with lower AUC than GPT-5.4 but a more balanced tradeoff between sensitivity and specificity. These differences matter because a model’s usefulness depends not on discrimination alone but on how its predictions translate into binary decisions in a given workflow. Representative true-positive, true-negative, false-positive, and false-negative pleural effusion cases are shown in Supplementary Figure S2.

These operating-profile differences would have very different consequences if such models were used in practice, although none of the evaluated models is validated or approved for any clinical use. A model with very low sensitivity, such as GPT-5.4, would miss most positive cases and could not serve as a standalone detector, whereas a model with very low specificity, such as Gemini 2.5 Pro, would generate a high false-positive burden. These scenarios illustrate why aggregate discrimination alone cannot indicate clinical readiness. Our results therefore argue against treating commercial MLLMs as interchangeable tools for chest radiograph interpretation. Even when evaluated on the same benchmark, they represent distinct operating strategies with different potential use cases and limitations. Prior studies evaluating earlier model generations on radiologic imaging tasks have similarly noted substantial variability in diagnostic behavior across commercial MLLMs [23,24,25,26]. For contextual comparison, CheXNeXt, a task-specific chest radiograph classifier evaluated against practicing radiologists, reported AUC values of 0.831 (95% CI, 0.790–0.870) for cardiomegaly, 0.924 (95% CI, 0.886–0.955) for edema, and 0.901 (95% CI, 0.868–0.930) for effusion. The corresponding radiologist AUC values were 0.888 (95% CI, 0.863–0.910), 0.910 (95% CI, 0.886–0.930), and 0.900 (95% CI, 0.876–0.921), respectively [9,27]. These values should not be interpreted as a direct head-to-head comparison with the present study, because CheXNeXt was evaluated on a different dataset, with different label definitions and a different reference standard. Nevertheless, they provide a useful numerical benchmark and indicate that zero-shot commercial MLLMs remain distinct from dedicated radiographic classifiers.

Sex-stratified differences were present but generally modest. Across cardiomegaly and pulmonary edema, AUC point estimates were consistently higher in male patients for all three models, whereas pleural effusion showed smaller and less directional subgroup differences. At the same time, most confidence intervals for sex-based differences overlapped zero, indicating that the observed effect sizes should be interpreted cautiously. The clearest subgroup deviations were seen not in global discrimination, but in specific operating metrics. Gemini 2.5 Pro showed a clear specificity difference for cardiomegaly, and Claude Opus 4.5 showed one for pulmonary edema, in both cases favoring male patients (Table 3). These findings suggest that subgroup disparities may manifest selectively in threshold-dependent behavior rather than uniformly across all performance measures [15,16].

From a clinical perspective, even modest subgroup differences deserve attention when they affect common and high-consequence findings such as cardiomegaly and pulmonary edema. Cardiomegaly is an important radiographic marker in cardiovascular disease assessment, and pulmonary edema is a key sign of acute heart failure and other urgent cardiopulmonary conditions. If model behavior differs systematically across patient groups, such differences could influence downstream workflows, especially if MLLMs are used for triage, prioritization, or preliminary interpretation. The present findings do not establish a single consistent pattern of bias across all tasks and models, but they do support the need for subgroup-aware auditing before any clinical use [19].

These results also have a methodological implication for the evaluation of MLLMs in medical imaging. Aggregate AUC alone is insufficient for judging model utility [1]. In this study, GPT-5.4 achieved the best AUC performance but would perform poorly as a standalone detector because of its very low sensitivity. Conversely, Gemini 2.5 Pro would capture most positive cases but at the cost of many false positives. Without reporting sensitivity and specificity alongside AUC, these clinically consequential differences would be obscured. The same principle applies to subgroup analysis: pooled performance can conceal variation in how models behave across patient strata. Benchmark studies of commercial MLLMs should therefore report both discrimination and operating characteristics, ideally with predefined subgroup analyses relevant to clinical use.

Several limitations should be acknowledged. First, the study used a single public dataset, and the findings may not generalize to other institutions, acquisition settings, or disease prevalences. Second, ground-truth labels were ultimately derived from radiology reports rather than direct image re-annotation for this study, although the use of VisualCheXbert-enhanced labels was intended to improve label quality [21]. Age balancing was performed using two broad strata (<70 and ≥70 years), which may not fully capture decade-specific age-related differences in radiographic appearance, particularly for findings such as cardiomegaly. Residual age-related confounding within these broad strata therefore cannot be excluded. Projection handling differed across cohorts: cardiomegaly and pleural effusion were restricted to PA views, whereas pulmonary edema included both PA and AP radiographs to reflect the acute-care distribution of edema imaging. Because AP projection can magnify the cardiac silhouette and alter the appearance of vascular redistribution and pulmonary congestion, projection-related effects may have influenced AUC and specificity estimates for pulmonary edema. This also limits direct comparability of performance estimates across the three pathology-specific cohorts. Third, the evaluation was restricted to zero-shot prompting and did not examine alternative prompt formulations, calibration strategies, or threshold optimization. Fourth, the models were assessed through commercial APIs, and their internal training data, update history, and optimization procedures are not transparent. A further transparency-related limitation is that GPT-5.4 was used by the authors for language editing and support in drafting and refining analysis code, while the same model was also one of the benchmarked systems. Although all analyses, code, and manuscript content were critically reviewed, tested, and verified by the authors, this overlap should be considered when interpreting the study and is therefore explicitly acknowledged. Furthermore, while MLLMs show high ranking performance, they remain susceptible to systematic interpretive errors and ‘hallucinations’ that are not yet fully captured by standard benchmark metrics [28]. Additionally, the confidence scores returned by the models were coarsely distributed across a small number of discrete levels rather than spanning the full 0–100 range continuously, as visualized in Supplementary Figure S1. These scores were subsequently transformed into model-derived probabilities for AUC-ROC estimation, but their discreteness may still limit the granularity of discrimination analysis and should be considered when interpreting differences between models, consistent with broader evidence that LLM self-reported confidence scores often show poor calibration despite reasonable discrimination [29]. Moreover, the cohorts were constructed with a balanced one-to-one ratio of positive and negative cases to ensure equal statistical power for the sex-stratified subgroup analyses. Because AUC-ROC is not mathematically dependent on class prevalence, the balanced design does not by itself inflate discrimination estimates. However, enriched case–control sampling may differ from routine practice in case mix and disease severity, and positive and negative predictive values cannot be inferred from the present balanced cohorts. In addition, radiographs were analyzed as downsampled JPEG images rather than full-resolution DICOM data viewed on diagnostic workstations, which may reduce the detectability of subtle findings and limits comparability with standard radiological reading conditions. Cases with co-occurring positive labels for the other two target findings were also excluded by design to isolate per-finding discrimination, which simplifies the task relative to clinical practice, where these findings frequently coexist. Finally, although sex-stratified differences were examined systematically, the study was not designed to determine the underlying mechanism of such differences, and the observed subgroup effects should therefore be interpreted descriptively rather than causally [30].

In conclusion, this benchmark shows that selected commercial MLLMs differ considerably in their diagnostic operating profiles for cardiopulmonary chest radiograph interpretation, even when tested under identical zero-shot conditions. GPT-5.4 showed the highest overall discrimination but an ultraconservative binary classification profile, Gemini 2.5 Pro prioritized sensitivity at the expense of specificity, and Claude Opus 4.5 showed the most balanced operating characteristics. Sex-stratified analyses revealed mostly modest but potentially relevant subgroup differences, particularly in specificity for selected model-pathology combinations. These findings caution against interpreting strong aggregate discrimination as evidence of clinical readiness. None of the evaluated models is currently suitable for autonomous chest radiograph interpretation, and benchmark reporting should extend beyond AUC alone to operating characteristics and prespecified subgroup analyses.

## Figures and Tables

**Table 1:** Cohort characteristics by pathology, disease status, and sex. Each pathology-specific cohort comprised 1500 frontal chest radiographs, balanced across four sex-by-label subgroups (male positive, male negative, female positive, female negative; *n* = 375 each). Age is reported as mean (SD).

Cohort	Status	Sex	*n*	Age, Mean (SD)
Cardiomegaly	Positive	Female	375	63.91 (17.70)
Cardiomegaly	Positive	Male	375	64.52 (16.45)
Cardiomegaly	Negative	Female	375	54.41 (17.71)
Cardiomegaly	Negative	Male	375	55.30 (17.26)
Edema	Positive	Female	375	59.55 (18.78)
Edema	Positive	Male	375	59.64 (18.69)
Edema	Negative	Female	375	57.85 (19.45)
Edema	Negative	Male	375	57.54 (18.94)
Pleural Effusion	Positive	Female	375	59.38 (16.42)
Pleural Effusion	Positive	Male	375	58.93 (16.22)
Pleural Effusion	Negative	Female	375	54.41 (17.71)
Pleural Effusion	Negative	Male	375	55.30 (17.26)

**Table 2:** Overall diagnostic performance of commercial multimodal large language models, pooled across sexes. Diagnostic performance is reported for GPT-5.4, Claude Opus 4.5, and Gemini 2.5 Pro on pathology-specific cohorts for cardiomegaly, pulmonary edema, and pleural effusion. Values are presented as AUC-ROC, sensitivity, and specificity with 95% bootstrap confidence intervals based on 1000 resampling iterations. Sensitivity and specificity were calculated from the models’ binary PRESENT/ABSENT classifications.

Pathology	Model	AUC-ROC (95% CI)	Sensitivity (95% CI)	Specificity (95% CI)
Cardiomegaly	GPT-5.4	0.859 (0.842–0.877)	0.293 (0.262–0.326)	0.977 (0.965–0.987)
Cardiomegaly	Claude Opus 4.5	0.742 (0.719–0.767)	0.648 (0.612–0.684)	0.731 (0.699–0.762)
Cardiomegaly	Gemini 2.5 Pro	0.760 (0.736–0.781)	0.916 (0.896–0.936)	0.449 (0.414–0.483)
Pulmonary Edema	GPT-5.4	0.836 (0.816–0.856)	0.043 (0.029–0.058)	0.997 (0.993–1.000)
Pulmonary Edema	Claude Opus 4.5	0.761 (0.736–0.785)	0.876 (0.850–0.897)	0.461 (0.427–0.495)
Pulmonary Edema	Gemini 2.5 Pro	0.745 (0.723–0.768)	0.973 (0.961–0.984)	0.241 (0.210–0.271)
Pleural Effusion	GPT-5.4	0.883 (0.866–0.899)	0.424 (0.390–0.459)	0.979 (0.968–0.988)
Pleural Effusion	Claude Opus 4.5	0.698 (0.671–0.723)	0.396 (0.360–0.431)	0.863 (0.837–0.885)
Pleural Effusion	Gemini 2.5 Pro	0.770 (0.747–0.794)	0.673 (0.641–0.709)	0.804 (0.775–0.830)

**Table 3:** Sex-stratified diagnostic performance and male–female performance differences. Performance is shown separately for male and female patients for each pathology and model. Sex-based differences are reported as male minus female values (Δ = Male − Female) for AUC-ROC, sensitivity, and specificity, with 95% bootstrap confidence intervals based on 1000 resampling iterations. Positive Δ values indicate higher performance in male patients, whereas negative Δ values indicate higher performance in female patients.

Pathology	Model	AUC Male (95% CI)	AUC Female (95% CI)	ΔAUC (95% CI)	Sens Male (95% CI)	Sens Female (95% CI)	ΔSens (95% CI)	Spec Male (95% CI)	Spec Female (95% CI)	ΔSpec (95% CI)
Cardiomegaly	GPT-5.4	0.865 (0.838–0.891)	0.852 (0.824–0.877)	+0.013 (−0.024 to +0.051)	0.304 (0.262–0.353)	0.283 (0.242–0.331)	+0.021 (−0.044 to +0.087)	0.971 (0.953–0.987)	0.984 (0.971–0.995)	−0.013 (−0.034 to +0.007)
Cardiomegaly	Claude Opus 4.5	0.756 (0.722–0.787)	0.729 (0.694–0.765)	+0.027 (−0.019 to +0.077)	0.661 (0.614–0.709)	0.635 (0.587–0.684)	+0.027 (−0.045 to +0.089)	0.747 (0.702–0.788)	0.715 (0.670–0.758)	+0.032 (−0.029 to +0.094)
Cardiomegaly	Gemini 2.5 Pro	0.774 (0.743–0.805)	0.744 (0.710–0.779)	+0.030 (−0.015 to +0.077)	0.915 (0.884–0.943)	0.917 (0.890–0.945)	−0.003 (−0.045 to +0.035)	0.493 (0.442–0.547)	0.405 (0.357–0.455)	+0.088 (+0.015 to +0.162)
Pulmonary Edema	GPT-5.4	0.852 (0.825–0.879)	0.821 (0.794–0.849)	+0.031 (−0.006 to +0.067)	0.053 (0.031–0.076)	0.032 (0.016–0.051)	+0.021 (−0.008 to +0.051)	1.000 (1.000–1.000)	0.995 (0.986–1.000)	+0.005 (+0.000 to +0.014)
Pulmonary Edema	Claude Opus 4.5	0.783 (0.748–0.813)	0.738 (0.703–0.771)	+0.045 (−0.003 to +0.089)	0.883 (0.850–0.914)	0.869 (0.836–0.902)	+0.013 (−0.035 to +0.058)	0.499 (0.451–0.548)	0.424 (0.376–0.474)	+0.075 (+0.004 to +0.147)
Pulmonary Edema	Gemini 2.5 Pro	0.749 (0.714–0.780)	0.742 (0.708–0.774)	+0.007 (−0.040 to +0.051)	0.968 (0.949–0.984)	0.979 (0.964–0.992)	−0.011 (−0.034 to +0.011)	0.264 (0.218–0.312)	0.219 (0.178–0.257)	+0.045 (−0.016 to +0.110)
Pleural Effusion	GPT-5.4	0.878 (0.853–0.902)	0.889 (0.863–0.912)	−0.011 (−0.045 to +0.022)	0.408 (0.359–0.458)	0.440 (0.389–0.487)	−0.032 (−0.100 to +0.033)	0.971 (0.952–0.987)	0.987 (0.974–0.997)	−0.016 (−0.037 to +0.004)
Pleural Effusion	Claude Opus 4.5	0.696 (0.659–0.732)	0.699 (0.663–0.733)	−0.002 (−0.053 to +0.050)	0.397 (0.347–0.446)	0.395 (0.344–0.447)	+0.003 (−0.070 to +0.071)	0.867 (0.831–0.900)	0.859 (0.823–0.894)	+0.008 (−0.043 to +0.058)
Pleural Effusion	Gemini 2.5 Pro	0.759 (0.725–0.791)	0.781 (0.751–0.813)	−0.022 (−0.068 to +0.023)	0.656 (0.607–0.706)	0.691 (0.646–0.736)	−0.035 (−0.099 to +0.030)	0.805 (0.766–0.847)	0.803 (0.764–0.842)	+0.003 (−0.050 to +0.057)

## Data Availability

The imaging data used in this study are publicly available through the CheXpert dataset, hosted by the Stanford Machine Learning Group (https://stanfordmlgroup.github.io/competitions/chexpert/, 21 May 2025). Access requires completion of a data use agreement. The VisualCheXbert labels used for ground-truth annotation are available at https://github.com/stanfordmlgroup/CheXbert, 21 May 2025. Cohort-level prediction outputs and analysis code are available from the corresponding author upon reasonable request.

## Supplementary Materials

The following supporting information can be downloaded at: https://www.mdpi.com/article/10.3390/diagnostics16132131/s1, Table S1: Standardized prompts used for zero-shot inference across all evaluated models; Table S2: Pairwise DeLong tests comparing AUC-ROC between models; Figure S1: Distribution of raw confidence scores by pathology and model; Figure S2: Representative pleural effusion cases illustrating consistent agreement across GPT-5.4, Claude Opus 4.5, and Gemini 2.5 Pro.
