# Supplementary material for: Benchmarking Multimodal Large Language Models for Cardiopulmonary Findings on Chest Radiographs: Sex-Stratified Discrimination and Operating Characteristics
Source: Diagnostics (Basel). 2026 Jul 7;16(13):2131. doi: 10.3390/diagnostics16132131 (PMC13361219; doi:10.3390/diagnostics16132131)
Supplement: Supplementary file 1 [file diagnostics-16-02131-s001.zip › diagnostics-4394941-supplementary.pdf]

Supplementary Table S1. Standardized prompts used for zero-shot inference across all evaluated models.

Prompt type	Prompt text
System instruction	You are an expert thoracic radiologist working in a research evaluation setting. You do not provide medical advice. Your task is binary visual classification from a frontal chest radiograph. Return strict JSON only.
Cardiomegaly prompt	Is cardiomegaly present on this frontal chest radiograph? Return ONLY strict JSON with EXACT keys: { "label": "PRESENT" | "ABSENT", "confidence": integer 0-100 } Confidence rubric (0-100): 90-100: very strong evidence; 75-89: strong evidence; 55-74: moderate evidence; 35-54: weak/equivocal evidence; 10-34: little or no convincing evidence. Choose the narrowest bin matching the evidence. Do not provide explanation, prose, or code fences.
Pulmonary edema prompt	Is pulmonary edema present on this frontal chest radiograph? Return ONLY strict JSON with EXACT keys: { "label": "PRESENT" | "ABSENT", "confidence": integer 0-100 } Confidence rubric (0-100): 90-100: very strong evidence; 75-89: strong evidence; 55-74: moderate evidence; 35-54: weak/equivocal evidence; 10-34: little or no convincing evidence. Choose the narrowest bin matching the evidence. Do not provide explanation, prose, or code fences.
Pleural effusion prompt	Is pleural effusion present on this frontal chest radiograph? Return ONLY strict JSON with EXACT keys: { "label": "PRESENT" | "ABSENT", "confidence": integer 0-100 } Confidence rubric (0-100): 90-100: very strong evidence; 75-89: strong evidence; 55-74: moderate evidence; 35-54: weak/equivocal evidence; 10-34: little or no convincing evidence. Choose the narrowest bin matching the evidence. Do not provide explanation, prose, or code fences.

Supplementary Table S2. Pairwise DeLong tests comparing AUC-ROC between models. Pairwise differences in AUC-ROC were assessed using paired, two-sided DeLong tests within each pathology-specific cohort. Holm-Bonferroni-adjusted p values account for the three pairwise model comparisons within each pathology.

Pathology	Comparison	AUC Model 1	AUC Model 2	ΔAUC	z	p	Holm-adjusted p
Cardiomegaly	GPT-5.4 vs Claude Opus 4.5	0.859	0.742	+0.117	10.46	<0.001	<0.001
Cardiomegaly	GPT-5.4 vs Gemini 2.5 Pro	0.859	0.760	+0.099	8.47	<0.001	<0.001
Cardiomegaly	Claude Opus 4.5 vs Gemini 2.5 Pro	0.742	0.760	-0.018	-1.30	0.194	0.194
Pulmonary edema	GPT-5.4 vs Claude Opus 4.5	0.836	0.761	+0.075	7.74	<0.001	<0.001
Pulmonary edema	GPT-5.4 vs Gemini 2.5 Pro	0.836	0.745	+0.091	8.49	<0.001	<0.001
Pulmonary edema	Claude Opus 4.5 vs Gemini 2.5 Pro	0.761	0.745	+0.016	1.40	0.162	0.162
Pleural effusion	GPT-5.4 vs Claude Opus 4.5	0.883	0.698	+0.186	15.78	<0.001	<0.001
Pleural effusion	GPT-5.4 vs Gemini 2.5 Pro	0.883	0.770	+0.113	10.60	<0.001	<0.001
Pleural effusion	Claude Opus 4.5 vs Gemini 2.5 Pro	0.698	0.770	-0.073	-5.45	<0.001	<0.001

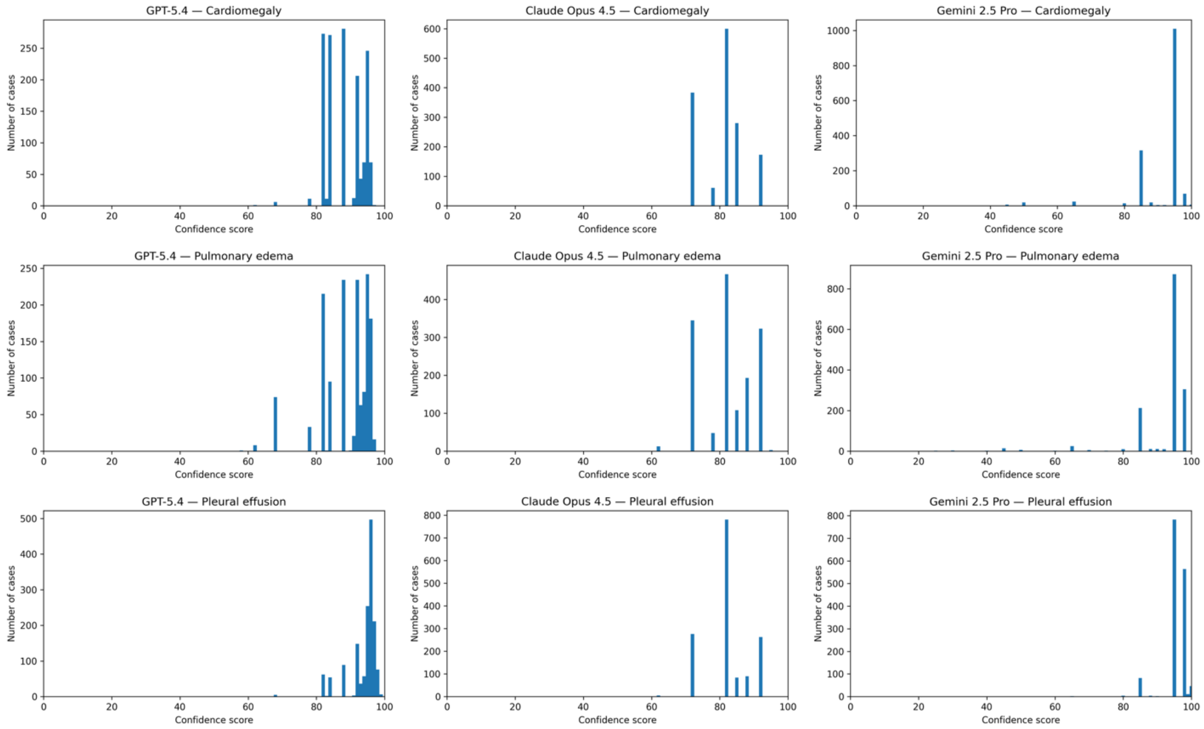

Supplementary Figure S1. Distribution of raw confidence scores by pathology and model. Histograms show the raw confidence scores returned by GPT-5.4, Claude Opus 4.5, and Gemini 2.5 Pro on a 0–100 scale for cardiomegaly, pulmonary edema, and pleural effusion.

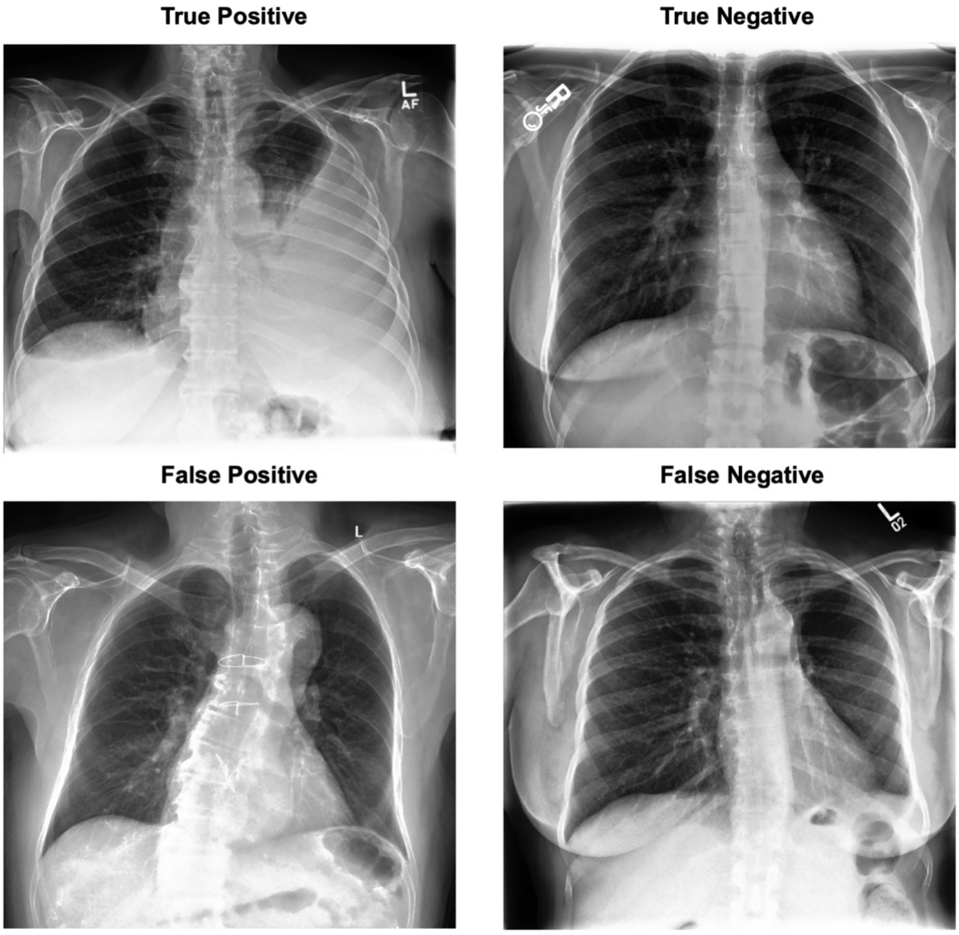

Supplementary Figure S2. Representative pleural effusion cases illustrating consistent agreement across GPT-5.4, Claude Opus 4.5, and Gemini 2.5 Pro. True positive (female, 65 years): pleural effusion correctly identified as present by all three models (GPT-5.4, 98%; Claude Opus 4.5, 92%; Gemini 2.5 Pro, 100%). **True negative** (female, 23 years): absence of pleural effusion correctly identified by all three models (GPT-5.4, 97%; Claude Opus 4.5, 92%; Gemini 2.5 Pro, 95%). **False positive** (male, 66 years): pleural effusion incorrectly reported as present by all three models despite ground-truth absence (GPT-5.4, 96%; Claude Opus 4.5, 82%; Gemini 2.5 Pro, 98%). **False negative** (female, 54 years): pleural effusion incorrectly reported as absent by all three models despite ground-truth presence (GPT-5.4, 92%; Claude Opus 4.5, 92%; Gemini 2.5 Pro, 95%).
